# Hepatitis C Virus Disease Progression in People Who Inject Drugs: Protocol for a Systematic Review and Meta-Analysis

**DOI:** 10.2196/resprot.4518

**Published:** 2015-06-08

**Authors:** Joan Combellick, Daniel J Smith, Ashly E Jordan, Holly Hagan

**Affiliations:** ^1^HCV Synthesis ProjectCollege of NursingNew York UniversityNew York, NYUnited States

**Keywords:** hepatitis C, people who inject drugs, HCV disease progression, fibrosis, hepatocellular carcinoma, cirrhosis, systematic review

## Abstract

**Background:**

Most hepatitis C virus (HCV) infections in the United States occur following non-sterile injection drug use. However, the majority of people who inject drugs (PWID) with chronic HCV are not currently receiving care.

**Objective:**

This paper presents our protocol for the systematic review and meta-analysis of data on the natural history of HCV among PWID and will inform modeling of the impact and cost-effectiveness of HCV management among this population. This study is conducted as part of the HCV Synthesis Project, which is funded to develop recommendations for HCV control strategies in the United States.

**Methods:**

This protocol describes the methods used for a systematic review and meta-analysis of published and unpublished data on the natural history of HCV among PWID including viral clearance, fibrosis progression, and the incidence of compensated cirrhosis (CC), decompensated cirrhosis (DC), hepatocellular carcinoma (HCC), and liver-related mortality.

**Results:**

Final results are anticipated by December 2016.

**Conclusions:**

Methods used for the synthesis of data on disease progression among HCV mono-infected PWID are presented. Data from the systematic review and meta-analysis will be used to inform simulations of the natural history of HCV and to model the effects of prevention and treatment strategies to reduce disease burden and the associated costs to society and individual patients.

## Introduction

In high-income countries, most cases of hepatitis C virus (HCV) infection are attributable to non-sterile drug injection [1,2]; the majority of chronically infected people who inject drugs (PWID) are not currently receiving care for their HCV infection [3]. Characteristics of both the virus and the population (PWID) complicate the diagnosis and management of chronic HCV infection [4]. Early infection with HCV is frequently asymptomatic, with the majority (75-85%) of those infected going on to develop chronic HCV infection [5,6]. The incubation period of HCV may last decades with symptoms only appearing after irreversible liver damage has occurred. The asymptomatic nature of HCV hampers early diagnosis, especially among those with limited or inconsistent access to health care, those uninsured or incompletely insured, and marginalized populations including PWID [7,8]. While new treatments for chronic HCV offer shorter treatment regimens with fewer side effects, high costs and concerns about reinfection after treatment limit the use and availability of these drugs.

An additional challenge to the study of the natural history of HCV is the disease’s long course towards clinically apparent liver sequelae and symptoms [9]. Disease outcomes may be underestimated because of competing mortality from events like drug overdose or co-morbidities like HIV [10,11]. Because disease duration often is estimated assuming that infection occurred at onset of drug injection rather than by observing the date of seroconversion, rates of disease progression also are underestimated [12,13]. Factors known to synergistically increase the rate of hepatic injury due to HCV—such as alcohol use—are difficult to systematically collect and often are not reported in studies on HCV natural history [13-16]. Finally, due to the challenges of outreach to a largely “hidden population” of PWID, study samples often are drawn from hospitalized patients and therefore may be biased toward greater disease severity [17].

Understanding HCV disease progression among PWID is critical to planning and allocating resources to control the HCV epidemic within this group. This paper presents our protocol for the systematic review and meta-analysis of data on the natural history of HCV among PWID and will inform modeling of the impact and cost-effectiveness of HCV management among this population. This study is conducted as part of the HCV Synthesis Project (see Hagan, Neurer, Jordan, Des Jarlais, Wu, Dombrowski, Khan, Braithwaite, and Kessler, 2014; Jordan, Des Jarlais, and Hagan, 2014) [18,19], which is funded to develop recommendations for HCV control strategies in the United States.

## Methods

### Design and Scope

The objective of this systematic review is to synthesize published and unpublished data on the natural history of HCV among PWID. HCV disease progression will be examined via the following outcomes: viral clearance, fibrosis progression rates, and the incidence of compensated cirrhosis (CC), decompensated cirrhosis (DC), hepatocellular carcinoma (HCC), and liver-related mortality. Factors contributing to progression towards each outcome (eg, the role of infection with genotype 3 in DC incidence) also will be examined.

### Criteria For Considering Reports

#### Inclusion and Exclusion Criteria

Reports will be included if: (1) the study sample is composed of participants who are chronically infected with HCV and report current or previous injection drug use; (2) original data on disease progression is presented; (3) at least 90% of the study sample is comprised of PWID; (4) findings are published or available after January 1, 1990; and (5) the study is conducted in upper-middle- or high-income countries (to insure comparability based on similar routes of infection, diagnosis, and treatment). Reports will be excluded if participants are co-infected with HIV, have received HCV treatment, or have undergone liver transplantation. Reports that include both mono- and co-infected participants will be accepted only if data from the HCV mono-infected sample is disaggregated.

#### Exposure Measures

The primary exposure of interest in this systematic review will be acute or chronic HCV infection. Adopting the European AIDS Treatment Network (NEAT) recommendations [20], the *preferred criteria* to measure acute exposure will be observed seroconversion, positive HCV antibody, and positive HCV RNA, or positive HCV RNA with a documented negative HCV RNA result in the previous 12 months. The *alternative criterion* for measuring exposure will be a single test result positive for anti-HCV antibody. The *preferred criterion* to measure chronic HCV infection will be documented persistent HCV RNA for at least 6 months [21]. The *alternative criterion* will be a statement in the report that all participants are chronically infected.

#### Outcome Measures

Primary outcomes examined will be the incidence and prevalence of spontaneous viral clearance, fibrosis, CC, DC or HCC, and liver-related mortality. To measure clearance of infection, the *preferred criterion* will be two or more consecutive undetectable HCV RNA test results separated by at least 6 months. *Alternative criterion* for clearance measurement will be a single negative RNA test result or an undetectable viral load.

The *preferred criterion* for measuring fibrosis and cirrhosis will be a liver biopsy staged according to the METAVIR, Ishak, Knodell, or Scheuer scoring systems, however, classification of cirrhosis using alternate diagnostic criteria or symptomatology also will be accepted. Data on fibrosis progression from noninvasive procedures such as FibroSURE and FibroScan will not be accepted. The criteria for measuring DC and HCC will include symptoms such as ascites, esophageal varices, and hepatic encephalopathy, and testing such as ultrasound.

To account for the underreporting of HCV as the underlying cause of death, the scope of the mortality outcome will include both HCV- and liver-related deaths among participants who have chronic HCV infection [22]. The *preferred criteria* for measuring liver-related mortality will be records from hospital databases and death registries.

### Search Strategy

Electronic searches will be conducted for literature published beginning January 1, 1990 using the following electronic databases: Ovid, Proquest, PubMed, and Web of Science. Search filters will include publication date, language (English only), and type of document (journal article). Manual searches of the reference lists of eligible reports, pertinent reviews and meta-analyses, and methodological papers will be reviewed. Abstracts from scientific conferences and presentations from study cohorts will also be screened for eligibility.

### Screening and Data Collection

Reports retrieved through the search strategy will be imported into Endnote X6 and duplicates will be removed. Two research assistants will perform the screening of abstracts and the extraction of data. Every abstract will be screened to determine its eligibility for inclusion in this study. Abstracts with any mention of PWID and any outcome of interest will be considered for inclusion, and the full-text report will be reviewed. For all potentially eligible reports, the full-text report will be reviewed by both research assistants to discern eligibility for inclusion. Reports meeting eligibility criteria will be coded. Reasons for exclusion will also be recorded.

Each research assistant will independently code the included reports using a coding tool that will be adapted from previous systematic reviews led by the principal investigator [23,24]. Coded data will be subsequently entered into a Microsoft Access database. The coding tool will include the following domains: citation information; study cohort, period, and location; study design; sampling, recruitment, testing, and statistical methodology; incidence or prevalence of spontaneous viral clearance, fibrosis, CC, DC, HCC, and liver-related mortality; rates of fibrosis progression; and, participant demographics, particularly risk factors associated with accelerated liver disease progression such as sex, race/ethnicity, and alcohol use. In the case of missing or inconsistent data in a report, the corresponding author will be contacted for additional information or clarification.

### Quality Assurance

#### Overview

Both of the research assistants involved in the project have graduate-level training in research methodology as well as additional training in HCV epidemiology and the methods of systematic review and meta-analysis. Pilot screening and coding tests will be conducted with and under the direction of the project director and principal investigator to assess inter-coder reliability and refine procedures for data extraction. The project director will evaluate all full-text reports excluded by the research assistants to corroborate their ineligibility. The research assistants will meet weekly during the coding process to compare their independent coding results. When consensus between the research assistants is not reached, the principal investigator and project director will be consulted; they will also review all coding to ensure accuracy and completion. Weekly staff meetings will provide a forum to discuss and resolve issues with data extraction. A study manual will guide the process and provide ongoing documentation of special cases and their resolution.

#### Report Quality and Critical Appraisal

All reports included for the systematic review will be assigned quality ratings based on an adapted version of the Quality In Prognosis Studies (QUIPS) tool [25]. The QUIPS tool will be modified to evaluate cross-sectional studies in addition to cohort studies and will be tailored to assess potential sources of bias in the included studies. Ratings of *high* (2), *moderate* (1), or *low* (0), will be assigned to judge the degree to which each study controls selection bias, confounding, and misclassification.

To account for the threat of selection bias, we will assess whether participant selection (eg, recruitment method or study location) is likely to alter the likelihood of observing the outcomes of interest. Comparability will be assessed in relation to the extent to which measures of association between prognostic factors and outcome appropriately adjust for the effects of confounding. Classification bias related to exposure and outcome will be evaluated including consistency in methods used to ascertain date of infection (eg, observed seroconversion vs date of first injection) and disease status. Misclassification of exposure (eg, acute vs chronic infection) will be evaluated by the criterion previously discussed.

### Data Analysis

This review will synthesize aggregate (report-level) data. Analysis will begin with an assessment for homogenous subsets within each outcome-specific set of reports. Given the expected variability in estimates across the reports, we will evaluate heterogeneity using the measures of Cochran’s *Q* (Der Simonian and Laird) and *I* ^2^, at each step of the analysis to distinguish between true variation of effects and variation due to other factors [26-28]. Data-based methods will be used to select covariates in multivariate models [29]. Effect size estimates will be combined using standard meta-analytic techniques in the form of pooled odds ratios and their 95% confidence intervals [30]. We will use random effects calculations whenever possible [31]. Potential moderator effects will be tested using meta-regression [32,33].

We anticipate having to modify the collected data in three different ways during data synthesis. First, effect measures reported as hazard ratios, risk ratios, or relative risks will be transformed into odds ratios using standard methods. Second, all data on fibrosis progression (including calculated fibrosis progression rates) will be standardized by converting all classification systems (eg, Ishak, Knodell or Scheuer) to METAVIR units [34]. If a report does not provide a progression rate, a progression rate of the stage-constant linear form will be calculated using one of two methods [35]: (1) for reports with participant-level data, the ratios of each participant’s METAVIR score to duration of infection in person-years will be summed, and the resulting mean will be used as the fibrosis progression rate [34,35], or (2) for reports with sample-level data, the fibrosis progression rate will be calculated as the ratio of participants’ cumulative METAVIR units to the estimated disease duration in person-years for the entire sample. Third, disease duration will be estimated using two different approaches: (1) infection starting from the date of first injection drug use, and (2) an adjusted duration calculated as 2 years after the date of first reported injection drug use. This second calculation is consistent with a prior meta-analysis of time to HCV infection among PWID [36].

## Results

The final results of this systematic review and meta-analysis are expected by December 2016.

## Discussion

This article presents a protocol for the systematic review and meta-analysis of disease progression among PWID with HCV mono-infection. Synthesized data on disease progression will be used to inform simulations of the natural history of HCV and to model the effects of prevention and treatment strategies to reduce disease burden and the associated costs to society and individual patients. This systematic review comes at a crucial time as new, better-tolerated, and more effective drugs become available to treat HCV infection. As the high cost of these new treatments spurs debate over resource allocation, it is crucial to understand the natural history of HCV disease among PWID, the largest population of HCV-infected persons in the United States.

## Multimedia Appendix 1

Reviewer Comments.

## Abbreviations

CC: compensated cirrhosis
DC: decompensated cirrhosis
HCC: hepatocellular carcinoma
HCV: hepatitis C virus
PWID: people who inject drugs
QUIPS: Quality in Prognosis Studies tool
