# Supplementary material for: Hepatitis C Virus Disease Progression in People Who Inject Drugs: Protocol for a Systematic Review and Meta-Analysis
Source: JMIR Res Protoc. 2015 Jun 8;4(2):e68. doi: 10.2196/resprot.4518 (PMC4526911; doi:10.2196/resprot.4518)
Supplement: Supplementary file 1 [file resprot_v4i2e68_app1.PDF]

SUMMARY STATEMENT
(Privileged Communication)

Release Date: 02/16/2013

PROGRAM CONTACT:
ELIZABETH LAMBERT Ph.D.
(301) 402-1933
el46i@nih.gov

Application Number: 1 R01 DA034637-01A1

Principal Investigator

HAGAN, HOLLY C. PHD

Applicant Organization: NEW YORK UNIVERSITY

Review Group: DIRH
Dissemination and Implementation Research in Health Study Section

Meeting Date: 02/08/2013
Council: MAY 2013
Requested Start: 07/01/2013

RFA/PA: PAR10-038
PCC: EB/EYL
Dual IC(s): AI

Project Title: Addressing Hepatitis C and Hepatocellular Carcinoma: Current and Future Epidemics
SRG Action: Impact Score: 11 Percentile: 2
Next Steps: Visit http://grants.nih.gov/grants/next_steps.htm
Human Subjects: 30-Human subjects involved - Certified, no SRG concerns
Animal Subjects: 10-No live vertebrate animals involved for competing appl.
Gender: 1A-Both genders, scientifically acceptable
Minority: 1A-Minorities and non-minorities, scientifically acceptable
Children: 1A-Both Children and Adults, scientifically acceptable
Clinical Research - not NIH-defined Phase III Trial

Project Year	Direct Costs Requested	Estimated Total Cost
1	447,684	696,738
2	435,888	678,379
3	497,902	774,893
4	421,841	656,518
TOTAL	1,803,315	2,806,528

ADMINISTRATIVE BUDGET NOTE: The budget shown is the requested budget and has not been adjusted to reflect any recommendations made by reviewers. If an award is planned, the costs will be calculated by Institute grants management staff based on the recommendations outlined below in the COMMITTEE BUDGET RECOMMENDATIONS section.

1R01DA034637-01A1 Hagan, Holly

RESUME AND SUMMARY OF DISCUSSION: This highly significant application will examine and synthesize the evidence on the effectiveness of different strategies for the prevention and control of hepatitis C (HCV) and its consequences while taking into account resource, epidemiologic and social network features. If successful, this research could help inform decision makers improve population health by providing guidance on the most effective set of interventions to control hepatitis C (HCV) infection in people who inject drugs and HIV positive men. This is an exceptional team of investigators with a strong track record of collaboration and record of publications. Most of the reviewers believed this revision to be extremely responsive to the prior reviews. The panel identified many strengths of the application and was very excited about the solid research design, strong preliminary studies, the use of agent based modeling approaches and the detailed dissemination plan. This was considered an impressive application from an exceptional team of investigators and enthusiasm was extraordinarily high. Overall there are no major weaknesses in this extremely strong application and reviewers concurred that the impact of this research on the field would likely be high.

DESCRIPTION (provided by applicant): Hepatitis C virus (HCV)-related deaths now exceed HIV-related deaths in the US. Throughout the world, HCV is hyperendemic in people who inject drugs (PWID). New outbreaks of acute HCV infection are unfolding in HIV-positive men who have sex with men (MSM) and in 15-24 year olds who have transitioned from abuse of prescription opioids to illicit opiate injection. In patients with chronic HCV infection, 20-25% will develop liver disease which may manifest as cirrhosis, liver failure or hepatocellular carcinoma (HCC). The prognosis for HCC is extremely poor, and HCV is the chief etiologic agent for this type of cancer. Recent discoveries in HCV prevention and treatment provide a great opportunity to reverse the trend toward increasing rates of HCV, HCV/HIV co-infection, and HCC. This study will use the methods of Implementation Science - research synthesis, mathematical modeling and simulation, and comparative effectiveness analyses - to determine how best to constitute a portfolio of interventions for the prevention and control of HCV and its consequences while taking into limited resources and underlying epidemiologic and social network features account. A dissemination plan will make extensive use of technology, including social media, and guidance from key stakeholders. These are our specific aims: 1. Synthesize evidence characterizing a) transition from misuse of prescription opioids to drug injection, b) HCV epidemiology and prevention for PWID and HIV+ MSM, and c) progression and treatment of HCV disease in these two groups, to derive best estimates to populate our HCV natural history and transmission models. 2. Use agent-based modeling to estimate the effects of scale-up of individual and combined prevention- and treatment-related interventions on HCV transmission and natural history in PWID and HIV+MSM. 3. Determine the combination of interventions for particular budget and epidemiologic scenarios that a) minimizes acute and chronic HCV infections, including HIV/HCV co-infection, b) prevents the greatest number of cases of HCV-related HCC and other serious sequelae, c) maximizes life expectancy and quality- adjusted life expectancy and d) reduces health disparities. 4. In collaboration with our Dissemination Advisory Board, apply an integrated knowledge-exchange approach to providing our target audiences (policymakers, public health and harm reduction practice communities, PWID and HIV+MSM) with the knowledge and tools to implement evidence-based HCV control strategies or reduce personal risk of infection and its consequences. The broad objective of this study is to provide an evidence base to guide allocation of scarce public resources in the US and other countries where HCV is principally transmitted among PWID. This will be accomplished by synthesizing, modeling and translating very recent developments in HCV epidemiology, prevention and treatment into practical tools to optimize population health.

PUBLIC HEALTH RELEVANCE: Statement The goal of this study is to help decision makers improve population health by providing guidance on the most effective set of interventions to control hepatitis C virus (HCV) infection in people who inject drugs and HIV- positive men who have sex with men. In the US, HCV is the main causal factor in hepatocellular carcinoma which has a very poor prognosis, and

HCV-related deaths now exceed deaths related to HIV. This research will synthesize evidence as to the effectiveness of different intervention strategies in terms of both individual benefit (preventing HCV infections in these populations, reducing HCC and other consequences of HCV), and in terms of societal benefit (reducing health care costs and impact on life expectancy).

CRITIQUE 1:

Significance: 1
Investigator(s): 1
Innovation: 1
Approach: 2
Environment: 1

Overall Impact: This is a resubmission that seeks to use agent-based modeling to simulate different combinations of intervention strategies in hopes of stemming the ongoing epidemic of hepatitis C virus (HCV) and its sequelae. They plan to conduct simulations of HCV transmission across different social networks and examine the long-term impact of different combinations of intervention strategies on HVC and HIV/HCV infection incidence. Given the epidemic of HCV and its sequelae, successful completion of this project has the potential to have a very high impact to the field. The revision has been extremely responsive to the previous reviewer comments and provides a compelling rationale for the need for the research and the methods used. The dissemination strategy that is now included greatly enhances the potential impact the research will have on both policy makers and the lives of those at risk for HCV related outcomes. Overall the project is well thought out and described and the investigators are experienced in both the content and the methods, leading to a high probability that the research could have a sustained and powerful impact on health care.

1. Significance:
Strengths
- Hepatitis C virus (HCV) is a major health concern requiring innovative interventions and this project provides the ability to rapidly identify issues and potential solutions through the use of simulation modeling.
- Community-based multi-components interventions are time-consuming and costly and there are simply too many combinations of components to test them in “live” trials, making this simulation approach extremely cost affective.
- The dissemination plan has the ability to reach many different stakeholders, including individuals at risk for HCV, in a timely fashion so choices can be rapidly made in integrating findings into policies and behaviors.

Weaknesses
- None noted

2. Investigator(s):
Strengths
- The principal investigator and investigative team are very strong with relevant experience and expertise to carry out the project.
- The principal investigator has conducted previous work on HCV and has considerable experience with the proposed methods.

Weaknesses
- None noted

3. Innovation:
Strengths

- The innovative and multi-faceted dissemination plan that goes beyond traditional academic strategies.
- The use of synthesized evidence from previous research in combination with simulation modeling is innovative.
- The research provides a thorough examination of different strategies for addressing the HCV epidemic.

Weaknesses

- None noted.

4. Approach:
Strengths

- The project builds on the previous work conducted by this team and is a next logical step.
- The team has experience in the use of the software and makes a compelling case that it is capable of simulating large social networks as proposed in this project.
- The use of agent based modeling is an efficient technique for accomplishing study aims and provides a relatively quick and cost-effective way to provide feedback that would otherwise take many years to accomplish.
- The project design is well thought out and logical, including the synthesis of existing evidence and the simulation of possible interventions.

Weaknesses

- None noted

5. Environment:
Strengths

- Excellent

Weaknesses

- None noted

Protections for Human Subjects:
Not Applicable (No Human Subjects)

- A letter from the institutional IRB is included confirming exemption from human subjects review.

Data and Safety Monitoring Plan (Applicable for Clinical Trials Only):
Not Applicable (No Clinical Trials)

Inclusion of Women, Minorities and Children:
G1A - Both Genders, Acceptable
M1A - Minority and Non-minority, Acceptable
C1A - Children and Adults, Acceptable

- While this project does not directly involve human subjects, the impact of the study results impacts both genders, minority and non-minority, as well as children.

Vertebrate Animals:
Not Applicable (No Vertebrate Animals)

Biohazards:
Not Applicable (No Biohazards)

Resubmission:

- The resubmission to this application has been extremely responsive to the concerns raised by the reviewers of the 1st submission. The dissemination plan is much more innovative and now

has the potential to more rapidly reach diverse stakeholders. The revised application also makes a strong case for and provides substantially more background information on meta-analyses.

Budget and Period of Support:
Recommend as Requested
Recommended budget modifications or possible overlap identified:

- Further justification is now provided for making this a four year project.

CRITIQUE 2:

Significance: 1
Investigator(s): 1
Innovation: 1
Approach: 2
Environment: 1

Overall Impact: This is a resubmission and the researchers were responsive to several of the comments in the previous review. They appropriately explained why they disagreed with some of the comments and these explanations were satisfactory.

- The proposed study will conduct simulations of HCV transmission across PWID and HIV+MSN networks and examine the impact of different combinations of intervention strategies on HCV and HIV?HCV infection incidence and related conditions.
- This is an innovative and important study that addresses a significant public health problem. The investigators are a highly experienced set of researchers that represent an impressive interdisciplinary team with the expertise to carry out the proposed study. The first study to comprehensively assess combination of HCV control strategies in the US.
- The use of computer simulation models is innovative.
- If successful, the study outcomes will guide program design and implementation for prevention and control of HCV, HIV/HCV co-infection, and hepatocellular carcinoma (HCC).
- The approach is clear and articulates a systematic approach using sequential steps to address aims and tasks including the description of relevant inputs and outputs.
- The inclusion of “constraints” that will be considered by the simulation (budget constraints, feasibility, and level of evidence) is a strength of this proposal and consistent with proposed efforts to make the information usable for decision-makers to enhance dissemination.
- Only minor weaknesses noted in the approach.

1. Significance:
Strengths

- Addresses an important problem of Hepatitis C virus infection and related morbidity and mortality. Proposes a study that could lead to important policy decisions concerning the potential impact of multicomponent interventions that target behavioral, biomedical, and structural issues in HCV control.
- Proposes an innovative modeling approach to examine how to constitute a portfolio of interventions to achieve the greatest effects given a particular budget and to compare the intervention bundle with alternate approaches.
- The proposed study, if successful, will guide program design and implementation for prevention and control of HCV, HIV/HCV co-infection, and hepatocellular carcinoma (HCC).

- Addresses an important public health problem particularly given the rise of HCV infection and associated conditions and given that combination prevention strategies may have additive or synergistic effects in reducing disease occurrence.
- Use of a Dissemination Advisory Board with multiple stakeholders is strength.

Weaknesses

- While the focus of this study is the two risk groups described above, it is likely that much of the information developed could advance knowledge in other groups. This is not discussed and could represent another element of potential significance of the proposed study.

2. Investigator(s):

Strengths

- Strong PI.
- Multidisciplinary team with expertise in the topic are, cost effectiveness and decision analytic modeling, epidemiology, social network analysis and comparative effectiveness research.

Weaknesses

- None noted.

3. Innovation:

Strengths

- The first study to comprehensively assess combination of HCV control strategies in the US.
- The use of computer simulation models is innovative and allows researchers to make use of existing data to better understand transmission, treatment, co-infection, and the potential impact of a various combinations of intervention approaches unencumbered by typical budget and time limitations present in traditional studies.
- The use of formal models of HCV virus dynamics in “agent-based dynamic network simulations” is an important innovative element of the proposed study.
- Addresses two emerging HCV epidemics (people transitioning to injection drug use and HIV+MSM) simultaneously.
- Will consider the effects of new DAA treatments for HCV infection and potential impact weighed against investments in HCV prevention.
- Will likely contribute to the development of novel methodological innovations represented by the proposed modeling plan.

Weaknesses

- It is unclear how the proposed research will “address existing disparities” and “assess how various control strategies may alleviate or exacerbate disparities.”

4. Approach:

Strengths

- Proposal describes a clear and systematic approach using sequential steps in the research plan to inform subsequent phases.
- Inputs and outputs of the activities described in the approach are clearly listed and relevant.
- The proposed evidence synthesis builds on and expands previous studies of the research team; the inclusion of “reviews of reviews” is a strength.
- The inclusion of “constraints” that will be considered by the simulation (budget constraints, feasibility, and level of evidence) is a strength of this proposal and consistent with proposed efforts to make the information usable for decision-makers to enhance dissemination.
- Cost considerations (both program and downstream costs) are comprehensive, appropriate and well-described.

- The Dissemination Plan described in aim 4 represents a coordinated effort with stakeholder participation to disseminate outcomes of the proposed study. Letters of support are included demonstrating willingness of stakeholders to participate in the project.
- The development of an online tool to assist stakeholders in using this information is a strength.

Weaknesses

- It is not clear how information from “reviews of reviews” will be weighed as compared to information from other reviews.
- No discussion on development steps for the online tool including usability assessments or other testing.

5. Environment:

Strengths

- Excellent. Proposed investigators have provided evidence of superb resources available to them and strong institutional support.

Weaknesses

- None noted.

Resubmission:

- The investigators were responsive to the previous review with several sections of the proposal substantially re-written.
- They appropriately explained why they disagreed with some of the comments and these explanations were satisfactory.
- Modified their approach to address the recommendation to use technology to enhance dissemination efforts.
- Added additional details related to cost as requested by the previous reviewers.
- Provided adequate justification concerning why the project needed to remain on a three year timeline.

CRITIQUE 3:

Significance: 1
Investigator(s): 2
Innovation: 1
Approach: 1
Environment: 1

Overall Impact: This revised submission proposes an innovative approach to an understudied and highly impactful/growing health problem (HCV). It is based on strong preliminary work by the investigators and uses innovative methods including simulation and agent-based modeling. The research team has been highly responsive to the original review, significantly enhancing the dissemination plan; providing more detailed explanation and rational for the modeling approach, and explaining in greater detail the approach to and use of the meta-analysis for developing recommendations for HCV control strategies. A minor concern is the lack of specification of the agent based modeling in terms of the assumptions needed for building such models. Both the study team and the environment are strong and they have addressed the previous human subjects concern.

Protections for Human Subjects:
Acceptable Risks and/or Adequate Protections

- The previous concern has been addressed

Data and Safety Monitoring Plan (Applicable for Clinical Trials Only):

Not Applicable (No Clinical Trials)

Inclusion of Women, Minorities and Children:
G4A - Gender Unknown, Acceptable
M4A - Minority Representation Unknown, Acceptable
C3A - No Children Included, Acceptable

Vertebrate Animals:
Not Applicable (No Vertebrate Animals)

Biohazards:
Not Applicable (No Biohazards)

Resubmission:
- very responsive to original critique

Resource Sharing Plans:
Acceptable

Budget and Period of Support:
Recommend as Requested

THE FOLLOWING RESUME SECTIONS WERE PREPARED BY THE SCIENTIFIC REVIEW OFFICER TO SUMMARIZE THE OUTCOME OF DISCUSSIONS OF THE REVIEW COMMITTEE ON THE FOLLOWING ISSUES:

PROTECTION OF HUMAN SUBJECTS (Resume): ACCEPTABLE

INCLUSION OF WOMEN PLAN (Resume): ACCEPTABLE

INCLUSION OF MINORITIES PLAN (Resume): ACCEPTABLE

INCLUSION OF CHILDREN PLAN (Resume): ACCEPTABLE

COMMITTEE BUDGET RECOMMENDATIONS: The budget was recommended as requested.

NIH has modified its policy regarding the receipt of resubmissions (amended applications). See Guide Notice NOT-OD-10-080 at http://grants.nih.gov/grants/guide/notice-files/NOT-OD-10-080.html.
The impact/priority score is calculated after discussion of an application by averaging the overall scores (1-9) given by all voting reviewers on the committee and multiplying by 10. The criterion scores are submitted prior to the meeting by the individual reviewers assigned to an application, and are not discussed specifically at the review meeting or calculated into the overall impact score. Some applications also receive a percentile ranking. For details on the review process, see http://grants.nih.gov/grants/peer_review_process.htm#scoring.

MEETING ROSTER

Dissemination and Implementation Research in Health Study Section
Healthcare Delivery and Methodologies Integrated Review Group
CENTER FOR SCIENTIFIC REVIEW
DIRH
February 08, 2013

CHAIRPERSON

BROWNSON, ROSS C, PHD
PROFESSOR OF EPIDEMIOLOGY
GEORGE WARREN BROWN SCHOOL OF SOCIAL WORK AND SCHOOL OF MEDICINE
WASHINGTON UNIVERSITY, ST. LOUIS
ST. LOUIS, MO 63108

MEMBERS

AMMERMAN, ALICE S, MPH, DRPH
PROFESSOR & DIRECTOR FOR HEALTH PROMOTION & DISEASE PREVENTION
DEPARTMENT OF NUTRITION
UNIVERSITY OF NORTH CAROLINA AT CHAPEL HILL
CHAPEL HILL, NC 27599

BARNASON, SUSAN ANN APRN, RN, PHD *
PROFESSOR
COLLEGE OF NURSING, LINCOLN DIVISION
UNIVERSITY OF NEBRASKA MEDICAL CENTER
LINCOLN, NE 68516

CRABTREE, BENJAMIN F, PHD
PROFESSOR AND RESEARCH DIRECTOR
DEPARTMENT OF FAMILY MEDICINE
ROBERT WOOD JOHNSON MEDICAL SCHOOL
UNIVERSITY OF MEDICINE AND DENTISTRY OF NEW JERSEY
SOMERSET, NJ 08873

EGEDE, LEONARD E, MD, MS
ALLEN H. JOHNSON ENDOWED CHAIR AND PROFESSOR OF MEDICINE
DIRECTOR, CENTER FOR HEALTH DISPARITIES RESEARCH
MEDICAL UNIVERSITY OF SOUTH CAROLINA
CHARLESTON, SC 29425

EMMONS, KAREN M, PHD
PROFESSOR
CENTER FOR COMMUNITY-BASED RESEARCH
DANA FARBER CANCER INSTITUTE
BOSTON, MA 02115

FERNANDEZ, MARIA EULALIA, PHD *
ASSOCIATE DIRECTOR
CENTER FOR HEALTH PROMOTION AND PREVENTION RESEARCH
SCHOOL OF PUBLIC HEALTH
UNIVERSITY OF TEXAS HEALTH SCIENCE CENTER
HOUSTON, TX 77030

GUSTAFSON, DAVID H, PHD
RESEARCH PROFESSOR AND DIRECTOR
CENTER FOR HEALTH ENHANCEMENT SYSTEMS STUDIES
UNIVERSITY OF WISCONSIN, MADISON
MADISON, WI 53726

HOUSTON, THOMAS K II, MPH, MD *
DIRECTOR
EHEALTH QUERI
BEDFORD VA MEDICAL CENTER
PROFESSOR, QUANTITATIVE HEALTH SCIENCES
UNIVERSITY OF MASSACHUSETTS MEDICAL SCHOOL
WORCESTER, MA 01655

HUNTER, SARAH , PHD *
BEHAVIORAL SCIENTIST
DEPARTMENT OF BEHAVIORAL SCIENCES
RAND CORPORATION
SANTA MONICA, CA 90407

KOGAN, STEVEN M, PHD *
ASSOCIATE PROFESSOR
CENTER FOR FAMILY RESEARCH
UNIVERSITY OF GEORGIA
ATHENS, GA 30602

MANDELL, DAVID S, SCD
ASSOCIATE PROFESSOR OF PSYCHIATRY AND PEDIATRICS
DEPARTMENT OF PSYCHIATRY AND PEDIATRICS
UNIVERSITY OF PENNSYLVANIA
PHILADELPHIA, PA 19104

RITZWOLLER, DEBRA P, PHD *
ECONOMIST, HEALTH SERVICES RESEARCHER
INSTITUTE FOR HEALTH RESEARCH
KAISER PERMANENTE COLORADO
LEGACY HIGHLANDS
DENVER, CO 80231

SHELLEY, DONNA R, MPH, MD *
DIRECTOR OF RESEARCH DEVELOPMENT
SCHOOL OF MEDICINE
NEW YORK UNIVERSITY
NEW YORK, NY 10016

STRAYHORN, GREGORY , MD, PHD *
PROFESSOR
DEPARTMENT OF FAMILY MEDICINE
MOREHOUSE SCHOOL OF MEDICINE
ATLANTA, GA 30308

VAN CLEAVE, JEANNE M, MD *
PEDIATRICIAN
CENTER FOR CHILD AND ADOLESCENT HEALTH RESEARCH AND POLICY
MASSACHUSETTS GENERAL HOSPITAL
BOSTON, MA 02114

WARRICK, CYNTHIA A, PHD *
PRESIDENT
SOUTH CAROLINA STATE UNIVERSITY
ORANGEBURG, SC 29117

ZGIBOR, JANICE CHRISTINE, PHD *
DIRECTOR DIABETES INSTITUTE
DEPARTMENT OF EPIDEMIOLOGY
UNIVERSITY OF PITTSBURGH
PITTSBURGH, PA 15261

MAIL REVIEWER(S)

DASANAYAKE, ANANDA P, BDS, PHD
PROFESSOR
DEPARTMENT OF EPIDEMIOLOGY
AND HEALTH PROMOTION
GRADUATE PROGRAM IN CLINICAL RESEARCH
NEW YORK UNIVERSITY COLLEGE OF DENTISTRY
NEW YORK, NY 10010

HAVRANEK, EDWARD , MD
PROFESSOR
DIRECTOR OF HEALTH SERVICES RESEARCH
AND STAFF CARDIOLOGIST
DENVER SCHOOL OF MEDICINE
UNIVERSITY OF COLORADO
DENVER, CO 80204

KHANNA, DINESH , MD
ASSOCIATE PROFESSOR OF MEDICINE
DIVISION OF RHEUMATOLOGY
DEPARTMENT OF INTERNAL MEDICINE
UNIVERSITY OF MICHIGAN
ANN ARBOR, MI 48109

LANDIS, CAROL A, FAAN, RN, BSN, DNSC
PROFESSOR
DEPARTMENT OF BEHAVIORAL NURSING
AND HEALTH SYSTEMS
SCHOOL OF NURSING
UNIVERSITY OF WASHINGTON
SEATTLE, WA 98195

SCHWARTZ, ROBERT A, MD
PROFESSOR AND DIRECTOR
DEPARTMENT OF DERMATOLOGY
NEW JERSEY MEDICAL SCHOOL
UNIVERSITY OF MEDICINE AND DENTISTRY, NEW JERSEY
NEWARK, NJ 07103

TOOSSI, ZAHRA , MD
PROFESSOR
DIVISION OF INFECTIOUS DISEASES
CASE WESTERN RESERVE UNIVERSITY
CLEVELAND, OH 44106

WILLIAMS, OLAJIDE , MD
ASSOCIATE PROFESSOR OF CLINICAL NEUROLOGY
ACUTE STROKE SERVICES
DEPARTMENT OF CLINICAL NEUROLOGY
COLUMBIA UNIVERSITY MEDICAL CENTER
NEW YORK, NY 10032

SCIENTIFIC REVIEW ADMINISTRATOR

BRONTE-TINKEW, JACINTA , PHD
SCIENTIFIC REVIEW OFFICER
CENTER FOR SCIENTIFIC REVIEW
NATIONAL INSTITUTES OF HEALTH
BETHESDA, MD 20892

GRANTS TECHNICAL ASSISTANT

AKINLOLU, ABDUL JABBAR O
LEAD EXTRAMURAL SUPPORT ASSISTANT
CENTER FOR SCIENTIFIC REVIEW
NATIONAL INSTITUTES OF HEALTH
BETHESDA, MD 20892

PIERCE, DEBRA
EXTRAMURAL SUPPORT ASSISTANT
CENTER FOR SCIENTIFIC REVIEW
NATIONAL INSTITUTES OF HEALTH
BETHESDA, MD 20892

SHEFFIELD-WRIGHT, REGINA
EXTRAMURAL SUPPORT ASSISTANT
CENTER FOR SCIENTIFIC REVIEW
NATIONAL INSTITUTES OF HEALTH
BETHESDA, MD 20892

* Temporary Member. For grant applications, temporary members may participate in the entire meeting or may review only selected applications as needed.

Consultants are required to absent themselves from the room during the review of any application if their presence would constitute or appear to constitute a conflict of interest.
